# A multimodal deep learning approach for mental health classification of university students: an intelligent early warning system

**DOI:** 10.3389/frai.2026.1679030

**Published:** 2026-06-04

**Authors:** Fu Li, Lei Wang, Wei Chen, Jian Zhang, Ying Huang, Chengxiao Jiang

**Affiliations:** 1 School of Automation, Hangzhou Dianzi University, Hangzhou, China; 2 Mental Health Education Center, Zhejiang Chinese Medical University, Hangzhou, China; 3 School of Cyberspace Security, Hangzhou Dianzi University, Hangzhou, China; 4 College of Information Engineering, Zhejiang University of Technology, Hangzhou, China

**Keywords:** applications in subject areas, data science applications in education, deep learning, mental health monitoring, natural language processing

## Abstract

**Introduction:**

Mental health issues among university students are becoming increasingly prominent, making an accurate and efficient mental state monitoring system a critical challenge in higher education management. Existing intelligent screening approaches mostly rely on single-modality data and are not tailored to counselor-student dialogue records or structured background information. Consequently, current systems struggle to provide reliable early warnings for high-risk students, especially under highly imbalanced risk-level distributions.

**Methods:**

This study develops a multimodal deep learning model, BCL (BERT-CNN-LASSO), which integrates counselor-student dialogue text with static background features (e.g., cumulative GPA, number of failed courses, financial aid status) to classify students into four ordered mental health risk levels for early warning. The model was evaluated on a curated real-world dataset of 600 university students collected from a counseling management system. BCL employs a BERT-based encoder for text representation, a dual-path CNN architecture for multi-scale feature extraction, and LASSO-based selection of static variables to enhance robustness under highly imbalanced risk distributions.

**Results:**

In the four-level mental health risk classification task, BCL achieved an overall accuracy of 0.752. At the category level, the model attained an accuracy of 0.897 for the highest-risk group (Level 1) and 0.967 for the lowest-risk group (Level 4), while maintaining a recall of 0.731 for Level 1, thereby reducing missed high-risk cases and limiting false alarms for low-risk students.

**Discussion:**

Comparative experiments demonstrate that each core component of BCL contributes to performance gains. These findings indicate that the proposed multimodal approach is a promising technical solution for intelligent early warning of university students' mental health problems.

## 1 Introduction

In recent years, mental health issues among university students have become a global concern (Pedrelli et al., 2015). Statistics indicate an increasing trend in the prevalence of psychological problems such as anxiety, depression, low self-esteem, and interpersonal sensitivity among college students (Copeland et al., 2021; Bantjes et al., 2022). Some studies suggest that mental health issues can lead to declined academic performance, reduced social adaptability, and even severe self-harming behaviors, including suicidal tendencies. This issue not only threatens students' personal growth and development but also poses significant negative impacts on family and social harmony and stability (Brown, 2018). Although traditional mental health assessment methods (such as questionnaires and regular psychological counseling) play crucial roles in identifying psychological problems, they often suffer from limitations such as high subjectivity, limited coverage, and insufficient real-time monitoring (Lin et al., 2017). Moreover, traditional methods are typically time-consuming in data collection and analysis, making it difficult to meet the demands of large-scale screening and timely intervention. Additionally, existing assessment methods often rely on students' initiative to seek help. However, due to various factors such as stigmatization of mental illness and lack of mental health literacy, many students with mental health issues lack the motivation to actively seek professional help, making it challenging to identify and intervene in potential mental health problems early (Jorm, 2012). Therefore, establishing an efficient and reliable monitoring and intervention mechanism for university students' mental health status is urgently needed.

With the rapid development of artificial intelligence and natural language processing technologies, researchers have begun to explore how these technologies can be utilized for automated detection of mental health issues. Current related research primarily focuses on analyzing social media text data, such as emotion analysis or depression detection from Twitter and Reddit user posts (Khafaga et al., 2023; Thelwall, 2017; Melcher et al., 2020). Monitoring mental health through textual data like tweets has proven effective in identifying vulnerable groups and helping detect potential mental health crises early (Paul and Dredze, 2011). However, the informal nature, metaphorical expressions, and cultural differences in social media texts increase analysis complexity. For instance, users might express suicidal tendencies through metaphors like “wanting to disappear,” which machine learning models might misinterpret due to lack of contextual understanding (Coppersmith et al., 2016). Expression differences across languages and cultural backgrounds (such as variations in suicide-related vocabulary between Chinese and English social media platforms) further complicate cross-cultural research (De Choudhury et al., 2016). Additionally, users may hide their true feelings for privacy concerns or present different personality traits on different platforms, making it difficult to accurately reflect their actual mental state (Skaik and Inkpen, 2020). Furthermore, existing methods mostly rely on single-modal data features, overlooking the significant value of multi-dimensional indicators such as background information and behavioral performance. Particularly in psychological counseling scenarios, the combined analysis of students' dialogue content and personal background information holds unique diagnostic value, yet relevant research remains scarce. Therefore, existing unimodal models face significant limitations when directly applied to mental state detection among Chinese university students, urgently requiring optimization through strategies including professional text analysis, multi-dimensional behavioral features, cultural semantic adaptation, and cross-modal data fusion to enhance the model's diagnostic validity and clinical applicability. These limitations indicate that current intelligent detection systems are not yet well suited for counselor-led early warning in university settings. In particular, there is a lack of multimodal models that jointly exploit counselor-student dialogue records and structured background features, explicitly target multi-level mental health risk stratification, and remain robust under the highly imbalanced risk distributions observed in real campuses.

In research based on student behavioral features, Liu et al. (2023) proposed a psychological anomaly detection early warning model combining data mining techniques with SVM. Their study collected six key attributes including students' basic information and academic performance through questionnaires, achieving high detection accuracy on a small-scale dataset. Luo and Li (2024) explored analysis methods based on multi-source behavioral data, incorporating multi-dimensional information such as students' consumption records, access control data, and academic performance. While these methods effectively utilize objective behavioral data, they lack attention to subjective experiences. Students' mental states often first manifest in their subjective feelings and emotional expressions (Abd Rahman et al., 2020). Relying solely on behavioral data makes it difficult to directly access students' inner thoughts and emotional changes, potentially causing models to overlook important psychological warning signals. Consequently, behavioral-feature-based systems alone cannot provide a sufficiently fine-grained or timely picture of students' psychological states, underscoring the need for multimodal early warning models that tightly couple counseling dialogue content with objective academic and background indicators.

To address these gaps, this paper proposes a multimodal deep learning model, BCL (BERT-CNN-LASSO), specifically designed for mental health early warning in Chinese universities. BCL jointly models counselor-student dialogue transcripts and structured static features, including academic performance and family socioeconomic status, to predict four ordered levels of mental health risk. At the architectural level, the model combines a BERT-Chinese encoder for domain-specific text representation, a dual-path CNN module for multi-scale feature extraction from dialogue records, and LASSO-based selection and compression of static vectors, together with an ensemble strategy across five counseling themes, thereby tailoring the representation learning process to the structure of real counseling data. The main contributions of this study are:

- Construction of a Multimodal Feature Fusion Framework: Innovatively designed the BCL model, achieving deep fusion of dialogue texts and static features through BERT-CNN dual feature extraction and LASSO feature selection.
- Development of Domain-Specific Data Analysis Methods: Unlike existing research mainly relying on non-professional data like social media, this study directly processes psychological counseling dialogue records, including records from five counseling themes and ten key background features, providing a more professional and reliable data foundation.
- Implementation of Precise Risk Level Classification: Employed a four-level classification system, offering more detailed differentiation of mental health issues compared to traditional binary classification methods, providing a basis for precise intervention.
- Provision of Interpretable Analysis Results: Through feature importance analysis, revealed key factors affecting university students' mental health, providing data support and theoretical guidance for mental health prevention and intervention work.

Overall, this study addresses the lack of counselor-oriented multimodal early warning systems that operate on real counseling records under highly imbalanced risk distributions, and demonstrates that such models can be trained and evaluated on routine university data in a way that is compatible with existing counseling workflows. These characteristics highlight the practical significance of the proposed model as a building block for scalable, data-driven mental health services in higher education.

## 2 Related work

### 2.1 Traditional methods for mental health monitoring

Traditional mental health monitoring methods primarily rely on questionnaires and face-to-face psychological counseling (Cruz-Gonzalez et al., 2025). Questionnaires are one of the common approaches; for example, Ebert et al. (2019) collected multiple predictors (such as gender, childhood abuse, academic failure) through questionnaires to construct mental health prediction models. However, questionnaire surveys are susceptible to respondents' subjective factors, leading to data authenticity bias, and can only reflect psychological states at specific time points, making real-time monitoring challenging.

While face-to-face psychological counseling can provide deeper understanding, it requires substantial human resources and is difficult to implement on a large scale (Dehbozorgi et al., 2025). Moreover, many students are reluctant to seek help actively due to stigmatizing attitudes toward mental illness (Jorm, 2012). Additionally, some studies have attempted to assess mental health status by observing students' daily behaviors. For instance, Morelli et al. (2017) discovered that students with different psychological traits exhibit varying degrees of centrality in social networks through analyzing behavioral patterns, yet this approach still struggles to comprehensively capture complex psychological states.

Therefore, traditional methods have inherent limitations in coverage, timeliness, and scalability for assessing college students' mental health, which underscores the need for more efficient and accurate automated monitoring approaches that can support large-scale, continuous early warning.

### 2.2 Text-based mental health monitoring

In recent years, with the advancement of natural language processing and deep learning technologies, text-based mental health monitoring methods have gradually become a research hotspot (Hoermann et al., 2017). These methods effectively identify individual psychological states, particularly common mental health issues such as depression and anxiety, through analyzing text content from social media, online forums, and psychological dialogue records (Madububambachu et al., 2024).

For example, Amanat et al. (2022) employed deep learning models (such as LSTM and CNN) to detect depression by analyzing Twitter users' textual data, showing that deep models can extract meaningful features from large volumes of unstructured text. Mathur et al. (2020) designed a natural language processing-based experiment utilizing time-series psycholinguistic cues to estimate suicide risk. More recent work has begun to incorporate transformer-based architectures and multilingual corpora for mental health detection, illustrating the potential of contextualized language models to capture subtle linguistic markers of distress in social media and forum posts (Khan et al., 2025; Patil and Gedhu, 2025). These studies collectively demonstrate the enormous potential of text data analysis in mental health monitoring.

Despite significant progress in text-based mental health monitoring methods, several challenges remain. First, the lack of unified and professional annotation standards for text data results in varying dataset quality. Additionally, the inherent subjectivity of text data means that considering text data alone is insufficient to ensure accuracy.

### 2.3 Multi-modal data-based mental health monitoring

With the development of big data and artificial intelligence technologies, mental health monitoring methods based on multi-modal data have become a research focus. These methods achieve more comprehensive capture of individual psychological states by fusing data from different sources, thereby improving monitoring accuracy and effectiveness (Asghar et al., 2024; Ding et al., 2024; Dayananda et al., 2024).

For instance, Ge et al. (2020) proposed a campus big data-based framework for monitoring university students' mental health, analyzing various behavioral data including internet usage records, dormitory access records, and cafeteria consumption records. Their research demonstrated that multi-source data fusion could effectively identify students with psychological issues and provide strong support for early intervention. Li and Zhou (2022) proposed a psychological emotion recognition algorithm based on multi-source data, using one-dimensional convolutional neural networks (1D-CNN) to mine students' online behavioral patterns and combining cafeteria consumption data to calculate anomaly scores for describing dietary differences. Through multi-dimensional data fusion, their research achieved a precision of 0.68 and a recognition rate of 0.75 in psychological problem identification tasks, providing systematic methodological support for schools to detect and intervene with students experiencing psychological issues. Mohr et al. (2017) utilized sensor data from smartphones and wearable devices, combined with machine learning algorithms, to extract multiple indicators including sleep, social context, emotion, and stress for mental health monitoring. More recent multimodal approaches integrate text, physiological signals, and behavioral traces to model complex mental states in both clinical and everyday settings (Guo et al., 2022; Xu et al., 2022). These studies demonstrate that multi-modal data fusion not only captures more information than any single modality but also enhances model robustness and interpretability.

However, despite the excellent performance of multi-modal data in mental health monitoring, several challenges remain. For example, the heterogeneity and complexity among different data sources make data fusion difficult. Furthermore, how to achieve efficient fusion of multi-modal data while ensuring model performance, and how to balance the contributions of different modal data are directions that require further exploration in this field.

## 3 Materials and methods

### 3.1 Data processing

#### 3.1.1 Data pre-processing

In the full counselor-management database, the prevalence of the four risk levels was highly imbalanced: Level 1 (0.63%), Level 2 (1.29%), Level 3 (1.62%), and Level 4 (96.46%). For model development and controlled comparison across levels, we constructed a class-balanced cohort of 600 students (150 per level). The 150 students in each level were obtained by simple random sampling from the corresponding level-specific pool in the database. No additional selection criteria were applied during sampling. All subsequent train, validation, and test splits were performed at the student level. This approach avoids subjective or manual filtering during cohort construction, while the balanced design mitigates class-imbalance effects in training and enables fair evaluation across risk strata. Following the four-level risk-stratification criteria summarized in Table 1 and expert annotation, each student was assigned to one of four ordered mental health risk levels (Levels 1-4, ranging from abnormal risk to low risk).

**Table 1:** Operational criteria for the four ordered mental health risk levels used as prediction targets.

Level	Expert-based criteria
Level 1 (abnormal risk)	Presence of a confirmed mental disorder and/or severe symptoms, accompanied by high comprehensive risk requiring urgent attention. Symptoms include: severe depressive/anxious symptoms with marked functional impairment; psychotic-like symptoms; high-risk behaviors; or explicit crisis signals (e.g., self-harm/suicidal ideation or intent).
Level 2 (high risk)	Confirmed mental disorder with relatively stable presentation *or* prominent symptoms of moderate severity, together with elevated comprehensive risk. Symptoms include: persistent moderate symptoms (e.g., depression/anxiety/stress-related reactions) with clear impact on daily functioning; or accumulating risk factors suggesting deterioration if not addressed.
Level 3 (moderate risk)	No confirmed severe disorder, but mild-to-moderate symptoms and/or early warning signs are present, with non-negligible comprehensive risk. Symptoms include: intermittent negative affect, academic stress with emerging functional impact, or mild psychosomatic complaints.
Level 4 (low risk)	Low or no risk: no evidence of severe disorder, and only mild, occasional, or situational difficulties are documented, with overall risk judged to be low.

The dialogue records were derived from periodic mental health interviews routinely conducted by university counselors. Interview implementation and documentation adhered to four institutional principles: timeliness, scientific objectivity, guidance and supportive care, and privacy protection. Interview content was recorded in a standardized format within the counselor management system to support subsequent student services and risk follow-up.

The classification of structured and unstructured features is summarized in Table 2. Each student record contains both structured and unstructured information. Structured variables include basic demographics and academic descriptors (e.g., major, grade, and gender). Unstructured data consist of counselor-documented dialogue records spanning five themes: ideological guidance, campus safety, academic guidance, career planning, and mental health. Each student has at least one record for each theme. Regarding text volume, each dialogue record contains on average 213 Chinese characters.

**Table 2:** Feature classification table.

Feature type	Feature items
Categorical features	Major, grade, gender, low-income family, Severely disabled family, single-parent family, orphan
Numerical Features	GPA, failed course count

The target variable is a four-level mental health risk label that jointly considers mental disorder morbidity and symptom severity and overall mental health risk. The level definitions were based on an evidence-informed risk stratification scheme adopted by 20 universities within the province and further refined through expert consensus. Labels were assigned through a structured panel process. For each case, at least two experts first reviewed the counselor records and proposed an initial level based on Table 1. Disagreements were resolved via panel discussion with reference to the documented symptom descriptions and risk signals; if consensus could not be achieved, a senior expert served as the final adjudicator.

The labeling guideline was finalized by a panel of seven experts (associate professors and PhD-level specialists) with expertise in mental health assessment and student psychological services. For each student, the experts reviewed multi-source information available in the counselor management system, including: (1) counselor-student interview records (text notes) collected during periodic mental health interviews; (2) documented psychological symptoms and risk signals described in the records; and (3) complementary background information recorded in the system (e.g., major/grade and relevant academic indicators). Labels were assigned according to the guideline, mapping each student to one of Levels 1-4.

For text data preprocessing, we employed the BERT tokenizer to tokenize the dialogue records. Formally represented as Equation 1:


$$\begin{array}{l} {\text{T}}_{s}={\text{BERT}}_{\text{tokenizer}}\left({\text{dialogue}}_{s}\right),\;s=1,2,3,\ldots \end{array}$$ (1)

where **T**_*s*_ represents the tokenization result of the *s*-th sample, with each token sequence padded or truncated to a uniform length of 64.

#### 3.1.2 Static vector feature extraction

In student early warning classification tasks, effective extraction and processing of static features significantly impact model performance (Mathes et al., 2017). Accordingly, we designed a multi-stage feature processing pipeline comprising four key steps: feature encoding, standardization, feature selection, and dimensionality reduction. Initially, based on the inherent nature of the data, we categorized features into categorical and numerical features:

For categorical features, considering their discrete and unordered nature, One-hot encoding was employed as shown in Equation 2:


$$\begin{array}{l} {\text{F}}_{\text{cat}}=\text{OneHot}\left({\text{X}}_{\text{cat}}\right)\in {\left\{0,1\right\}}^{n\times d_{\text{cat}}} \end{array}$$ (2)

where *n* represents the number of samples, and *d*_cat_ represents the dimensionality of transformed categorical features. One-hot encoding converts each categorical feature into a binary vector, ensuring no erroneous ordinal relationships are introduced between features.

For numerical features, Standard Scaler was used for standardization to eliminate scale differences between features, initializing all features to have equal contribution levels to the model as shown in Equation 3:


$$\begin{array}{l} {\text{F}}_{\text{num}}=\frac{{\text{X}}_{\text{num}}-\mu }{\sigma } \end{array}$$ (3)

where μ and σ represent the mean and standard deviation of each feature, respectively.

The feature selection phase employs Lasso-regularized multinomial logistic regression for feature importance assessment. For K-class classification (K = 4 in this study), the LASSO model optimizes by minimizing the following objective function as shown in Equation 4:


$$\begin{array}{l} L\left(\beta \right)=\underset{\beta }{\min}\left\{-\overset{n}{\underset{i=1}{\sum }}\overset{K}{\underset{k=1}{\sum }}y_{ik}\log\left(p_{ik}\right)+\lambda \overset{p}{\underset{j=1}{\sum }}|\beta_{k,j}|\right\} \end{array}$$ (4)

where *p*_*ik*_ represents the predicted probability of the *i*-th sample belonging to class *k*, calculated through the softmax function as shown in Equation 5:


$$\begin{array}{l} p_{ik}=\frac{\exp\left(\beta_{k}^{T}x_{i}\right)}{\sum_{l=1}^{K}\exp\left(\beta_{l}^{T}x_{i}\right)} \end{array}$$ (5)

Here, *y*_*ik*_ is the one-hot encoding of labels (1 if the *i*-th sample belongs to class *k*, 0 otherwise), *x*_*i*_ is the feature vector of the *i*-th sample, β_*k*_ is the coefficient vector for class *k*, and λ is the regularization parameter controlling model sparsity. The optimal λ value is selected through 5-fold cross-validation, achieving automatic feature selection while maintaining model predictive‘performance.

Feature importance is calculated through the absolute values of model coefficients, as shown in Equation 6:


$$\begin{array}{l} {\text{Importance}}_{j}=\overset{K}{\underset{k=1}{\sum }}|\beta_{j,k}| \end{array}$$ (6)

where β_*j,k*_ represents the weight coefficient of the *j*-th feature for the *k*-th class. Lasso regularization achieves two key objectives by adding an L1 norm penalty term to the objective function: feature sparsification, where L1 norm characteristics cause certain feature weights to be exactly zero, automatically achieving feature selection; and overfitting control, improving model generalization ability by limiting model complexity (Ranstam and Cook, 2018).

To further reduce feature space dimensionality, PCA dimensionality reduction is applied to the selected feature set (Maćkiewicz and Ratajczak, 1993), as formalized in Equation 7:


$$\begin{array}{l} {\text{F}}_{\text{final}}=\text{PCA}\left({\text{F}}_{\text{selected}}\right),\text{ s.t. }\frac{\sum_{i=1}^{k}\lambda_{i}}{\sum_{i=1}^{p}\lambda_{i}}\geq 0.7 \end{array}$$ (7)

where **F**_selected_ is the feature matrix after Lasso selection, λ_*i*_ is the eigenvalue of the *i*-th principal component, and *k* is the feature dimensionality after reduction. The constraint ensures retention of 70% of the original data's variance information, maximally preserving crucial data information while reducing dimensionality.

### 3.2 Proposed model

**Figure 1:**
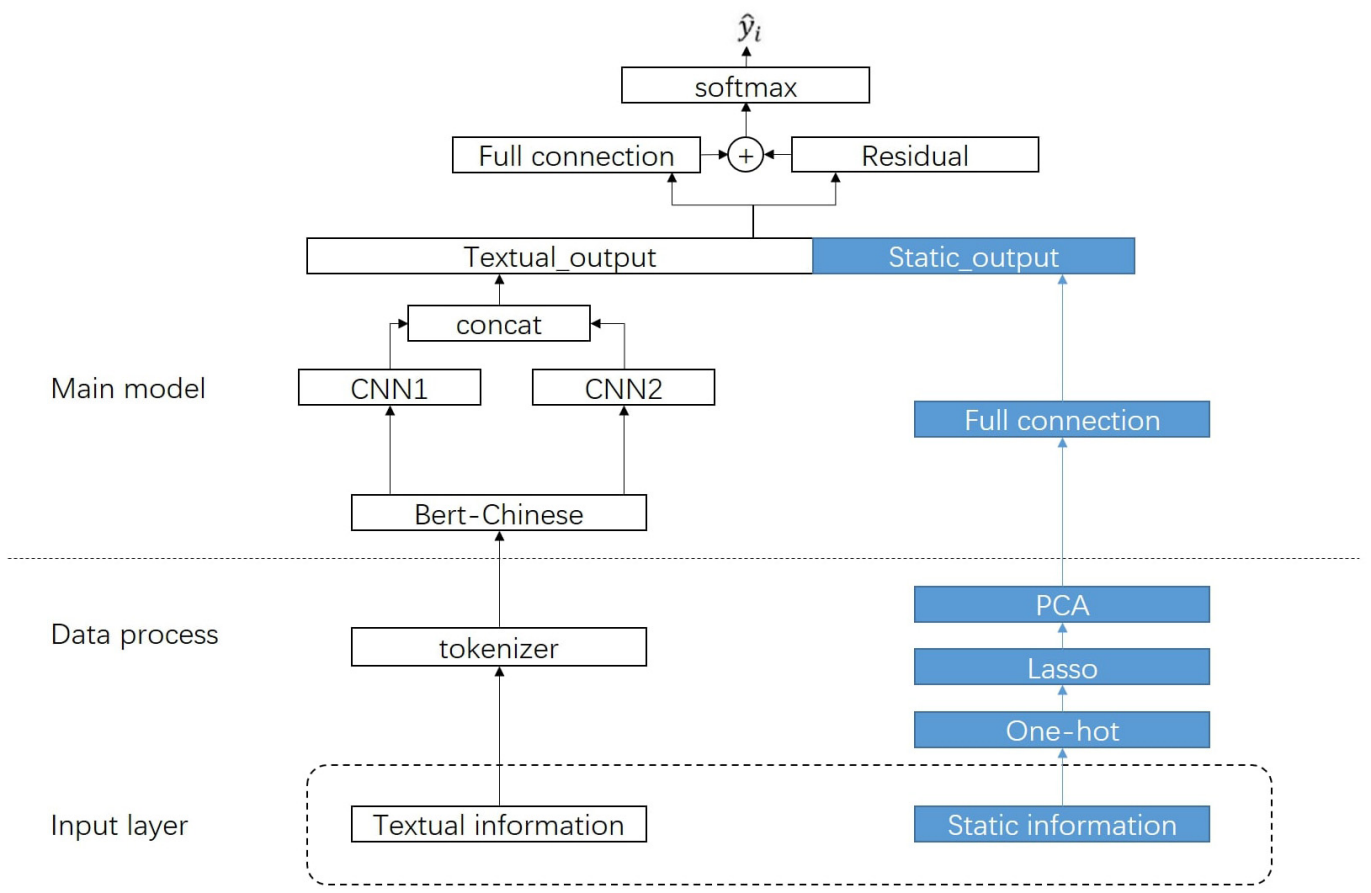
Model architecture.

To address the complex multimodal task of student mental health status classification, we propose the BCL (BERT-CNN-Lasso) model. This model combines BERT's text feature extraction capabilities, CNN's multi-scale feature learning advantages, and Lasso feature engineering's data denoising and selection benefits. The model adopts an ensemble learning strategy consisting of five category-specific base classifiers, where each dialogue theme is modeled by an independent BCL network with the same architecture as shown in Figure 1 For a given student sample, each base classifier first aggregates predictions across multiple dialogue records to obtain a theme-level probability vector. The final prediction is then produced by an adaptive weighted integration of the five theme-level outputs.

#### 3.2.1 Text feature extraction module

Considering the rich semantic information contained in dialogue records, we first employ the pre-trained BERT variant BERT-Chinese model as the base encoder. Through its bidirectional encoder architecture, BERT can effectively capture semantic variations of words in different contexts. Compared to traditional word embedding methods like Word2Vec or GloVe, BERT-Chinese not only possesses stronger contextual awareness but also leverages knowledge from large-scale Chinese corpus pre-training to enhance language understanding. Additionally, the WordPiece-based tokenization strategy enables better processing of Chinese text semantic units. The mathematical expression for text feature extraction is Equation 8:


$$\begin{array}{l} {\text{H}}_{\text{BERT}}=\text{BERT}\left({\text{T}}_{i}\right)\in {\mathbb{R}}^{768} \end{array}$$ (8)

where **T***i* represents the input text sequence, producing a 768-dimensional feature vector**H**_BERT_ after BERT encoding.

To further enhance text feature representation capability, we designed a dual-path CNN structure. CNN excels at processing grid-structured data (such as text sequences or images), with its core idea being automatic multi-scale feature learning through local receptive fields and weight sharing mechanisms. In text processing, CNN effectively captures local semantic dependencies (such as phrases or grammatical structures) between words through sliding convolution kernels of different sizes over word embedding sequences (Alzubaidi et al., 2021). We achieve multi-scale feature extraction through parallel one-dimensional convolution layers with different kernel sizes, as formalized in Equation 9:


$$\begin{array}{l} {\text{C}}_{r}=\sigma ({\text{H}}_{\text{BERT}}*{\text{W}}_{r}+{\text{b}}_{r}),\;r\in \left\{3,5\right\}, \end{array}$$ (9)

where ${\text{W}}_{r}\in {\mathbb{R}}^{r\times d\times F_{r}}$ is the convolution kernel with window size *r*, *F*_*r*_ is the number of filters, * denotes one-dimensional convolution over the sequence dimension, **b**_*r*_ is a bias term, and σ(·) is a non-linear activation function (ReLU). The resulting feature maps ${\text{C}}_{r}\in {\mathbb{R}}^{L_{r}\times F_{r}}$ capture local semantic patterns at different n-gram scales. This design not only simultaneously obtains text features at different granularities but also significantly improves processing efficiency through CNN's parallel computing characteristics. Compared to Recurrent Neural Networks (RNN), this structure substantially reduces computational complexity while maintaining performance.

In the feature fusion stage, CNN-extracted features are processed through max-pooling operations and merged with static features. Max-pooling operations preserve the most significant feature activations while providing robustness to positional variations of key information in the text. The feature fusion process can be represented as Equation 10:


$$\begin{array}{l} {\text{F}}_{c}=\left[\text{MaxPool}\left({\text{C}}_{1}\right);\text{MaxPool}\left({\text{C}}_{2}\right);{\text{F}}_{f}\right] \end{array}$$ (10)

where $\text{MaxPool}\left({\text{C}}_{k}\right)\in {\mathbb{R}}^{F_{k}}$ denotes a global max-pooling operation over the temporal dimension of **C**_*k*_, and ${\text{F}}_{f}\in {\mathbb{R}}^{q}$ is the PCA-reduced static feature vector. The concatenated vector ${\text{F}}_{c}\in {\mathbb{R}}^{F_{3}+F_{5}+q}$ thus forms a joint representation of dialogue content and static context for a single dialogue record. Max-pooling operations compress CNN outputs into fixed-dimensional feature representations, enabling uniform processing of input sequences of varying lengths. This fusion strategy effectively integrates information from different modalities, enabling the model to utilize both students' textual interaction information and static feature information for more accurate classification decisions.

#### 3.2.2 Classification module

The classification module adopts a residual-connected multilayer perceptron structure. First, feature transformation is performed through multilayer perceptron and regularization components, as shown in Equation 11:


$$\begin{array}{l} \text{H}=\text{Dropout}\left(\text{ReLU}\left(\text{LayerNorm}\left({\text{W}}_{1}{\text{F}}_{c}+{\text{b}}_{1}\right)\right)\right) \end{array}$$ (11)

where **W**_1_ and **b**_1_ are the weight matrix and bias vector of the first layer respectively. The final classification probabilities are computed with residual connections, as defined in Equation 12:


$$\begin{array}{l} \text{y}=\text{softmax}\left({\text{W}}_{2}\text{H}+{\text{W}}_{r}{\text{F}}_{c}\right) \end{array}$$ (12)

where **W**_2_ is the main path weight matrix and **W**_*r*_ is the residual path weight matrix. The introduction of residual connections effectively mitigates the vanishing gradient problem in deep networks, ensuring smooth information flow. The combined use of layer normalization and Dropout mechanisms further enhances model stability and generalization capability.

#### 3.2.3 Loss function and optimization strategy

During model training, we designed a two-stage optimization strategy, including base model training and ensemble weight optimization. For each base model, cross-entropy loss function serves as the primary optimization objective Equation 13:


$$\begin{array}{l} L_{\text{base}}=-\overset{N}{\underset{n=1}{\sum }}\overset{C}{\underset{c=1}{\sum }}y_{n,c}\log\left({ŷ}_{n,c}\right) \end{array}$$ (13)

where *N* is the batch size, *C* is the number of classes (4 in this study), *y*_*i,c*_ is the true label (0 or 1) for sample *i* and class *c*, and ŷ_*i,c*_ is the model's predicted probability for sample *i* belonging to class *c*.

For multiple dialogue records of each sample, we adopt a confidence-based weighted averaging strategy, as formalized in Equation 14:


$$\begin{array}{l} {ŷ}_{\text{sample}}=\overset{M}{\underset{d=1}{\sum }}w_{d}{ŷ}_{d},\;w_{d}=\frac{\exp\left(s_{d}/T\right)}{\sum_{k=1}^{M}\exp\left(s_{k}/T\right)} \end{array}$$ (14)

where ${\hat{\text{y}}}_{d}$ denotes the predicted class-probability vector (after softmax) for the *d*-th dialogue record of the sample, *M* is the number of dialogue records for the sample, *s*_*d*_ is the confidence score of the *d*-th prediction, and *T* is the temperature parameter (set to 0.5).

**Figure 2:**
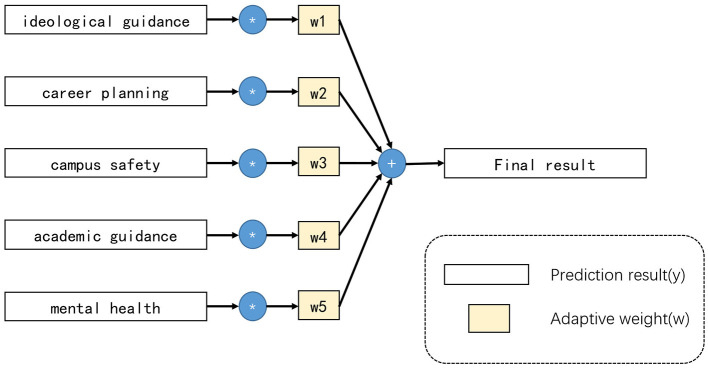
Ensemble architecture.

In the ensemble stage, we designed an adaptive weight ensemble strategy to fuse the outputs of models corresponding to five dialogue categories. The ensemble prediction is calculated as Equation 15:


$$\begin{array}{l} {ŷ}_{f}=\overset{5}{\underset{m=1}{\sum }}w_{m}{ŷ}_{m} \end{array}$$ (15)

where ŷ_*m*_ represents the prediction result of the *m*-th base model (theme), *w*_*i*_ is the corresponding adaptive weight coefficient, and ŷ_*f*_ is the final result for this student. This process is shown in Figure 2.

The objective function for ensemble weight optimization is Equation 16:


$$\begin{array}{l} L_{t}=L_{\text{CE}}\left(y,{ŷ}_{f}\right)+\lambda |\Theta {|}_{2}^{2} \end{array}$$ (16)

where λ is the regularization coefficient (set to 0.05). Weight optimization uses the Adam optimizer with a learning rate of 1e-3. For base model training, we employ the AdamW optimizer with an initial learning rate of 1e-4 and weight decay coefficient λ. We also incorporate the ReduceLROnPlateau strategy, reducing the learning rate to 0.8 times its original value when validation performance stagnates. Additionally, we implemented an early stopping mechanism, halting training when validation set performance shows no improvement for 5 consecutive epochs, effectively preventing overfitting.

From a modeling perspective, the BERT-CNN-LASSO architecture influences the mental health classification process in several complementary ways. First, the BERT-Chinese encoder provides contextualized token representations that are sensitive to subtle linguistic markers of distress, which is critical for counseling dialogues where high-risk signals may be expressed indirectly or with euphemistic wording. Second, the dual-path CNN module aggregates these contextual embeddings over multiple n-gram windows, allowing the model to capture both short phrases (e.g., explicit symptom descriptions) and longer local patterns (e.g., recurring concerns about academic failure or family conflict) with efficient parallel computation. Third, the LASSO-based static feature pipeline denoises and compresses heterogeneous background variables into a low-dimensional vector that emphasizes the most predictive academic and socioeconomic indicators, thereby stabilizing the multimodal fusion and reducing overfitting on a small sample size. Finally, the theme-specific ensemble structure aligns the model with the organization of real counseling workflows, ensuring that information from different counseling themes can contribute with different strengths to the final risk estimate. Together, these design choices enable BCL to improve both the recall for highest-risk students and the precision for low-risk students, as later confirmed by the comparative and ablation experiments.

## 4 Experimental results and analysis

### 4.1 Experimental setup

The experimental dataset comprises mental health records of 600 university students, each containing psychological counseling transcripts across five themes and ten personal background features (including hometown, GPA, etc.). The data was categorized into four risk levels by expert annotations. Experiments were conducted using PyTorch 1.9 (Meta Platforms, Inc., Menlo Park, California, USA) framework on NVIDIA RTX 3090 GPUs. Training parameters included an AdamW optimizer with initial learning rate of 1e-4, batch size of 32, maximum training epochs of 50, and early stopping mechanism to prevent overfitting.

### 4.2 Evaluation metrics

To comprehensively evaluate model performance, this study employs two accuracy calculation methods along with other evaluation metrics. The overall accuracy reflects the model's correct prediction proportion across all samples as shown in Equation 17:


$$\begin{array}{l} Acc_{\text{overall}}=\frac{\sum_{i=1}^{4}Ncorrect_{i}}{N} \end{array}$$ (17)

where *Ncorrect*_*i*_ denotes the number of correctly predicted samples in class *i*, and *N* represents the total sample size. For each class *i*, specific performance metrics are calculated as follows (Equations 18–21):


$$\begin{array}{l} Acc_{i}=\frac{TP_{i}+TN_{i}}{TP_{i}+TN_{i}+FP_{i}+FN_{i}} \end{array}$$ (18)

$$\begin{array}{l} Precision_{i}=\frac{TP_{i}}{TP_{i}+FP_{i}} \end{array}$$ (19)

$$\begin{array}{l} Recall_{i}=\frac{TP_{i}}{TP_{i}+FN_{i}} \end{array}$$ (20)

$$\begin{array}{l} F1_{i}=\frac{2\times Precision_{i}\times Recall_{i}}{Precision_{i}+Recall_{i}} \end{array}$$ (21)

where *TP*_*i*_, *TN*_*i*_, *FP*_*i*_, and *FN*_*i*_ respectively represent true positives, true negatives, false positives, and false negatives for class *i*. Notably, the class-specific accuracy *Acc*_*i*_ simultaneously considers both correct identification of target class (*TP*_*i*_) and non-target classes (*TN*_*i*_), differing from the calculation methodology of overall accuracy.

### 4.3 Overall performance analysis

The model achieved an overall accuracy of 0.7519 on the test set, with performance by category shown in Table 3.

**Table 3:** Classification performance by attention level.

Attention level	Accuracy	Precision	Recall	F1 score
Level 1	0.8967	0.7209	0.7305	0.7256
Level 2	0.8185	0.6378	0.6856	0.6608
Level 3	0.8219	0.6730	0.6528	0.6627
Level 4	0.9667	0.9583	0.9270	0.9424

The experimental results demonstrate that the model performs exceptionally well for Level 4, achieving an accuracy of 0.9667 with all other metrics above 0.92. This result indicates the model's ability to accurately identify students with stable mental states, effectively reducing false alarm rates and improving the early warning system's credibility. This is particularly significant for avoiding unnecessary interventions and resource waste.

In terms of high-risk group identification, the model achieved an accuracy of 0.8967 and recall of 0.7305 for Level 1. Performance metrics for Level 1 are notably better than those for Levels 2 and 3. This performance distribution aligns well with the practical requirements of psychological early warning systems-prioritizing accurate identification of high-risk students. Particularly, the 0.7305 recall rate indicates the model's capability to capture most cases requiring Level 1 attention, crucial for preventing serious psychological issues. From an operational perspective, early-warning systems are typically deployed as decision-support tools rather than final diagnostic instruments. In such settings, the objective is to prioritize a manageable subset of students for timely screening and follow-up under limited counseling capacity. A recall of 0.7305 suggests that the system can substantially reduce the probability of missing high-risk cases compared with unguided or purely rule-based triage, while maintaining a moderate false-alarm burden. Importantly, the proposed system is designed to be integrated with existing counseling workflows, where flagged cases trigger secondary human assessment to balance intervention costs and safety.

However, there remains room for improvement in classifying moderate risk levels. The F1 scores for Levels 2 and 3 are 0.6608 and 0.6627 respectively, lower than those of the extreme categories. This performance disparity primarily stems from three aspects: moderate-risk students often exhibit characteristics of multiple levels simultaneously, such as academic pressure coexisting with mild emotional fluctuations, increasing classification difficulty; compared to Level 1's clear warning signals and Level 4's stable presentation, moderate risk level assessment criteria are relatively ambiguous with unclear boundaries; students in these categories may use more implicit or indirect expressions in dialogue texts, reducing feature salience.

**Figure 3:**
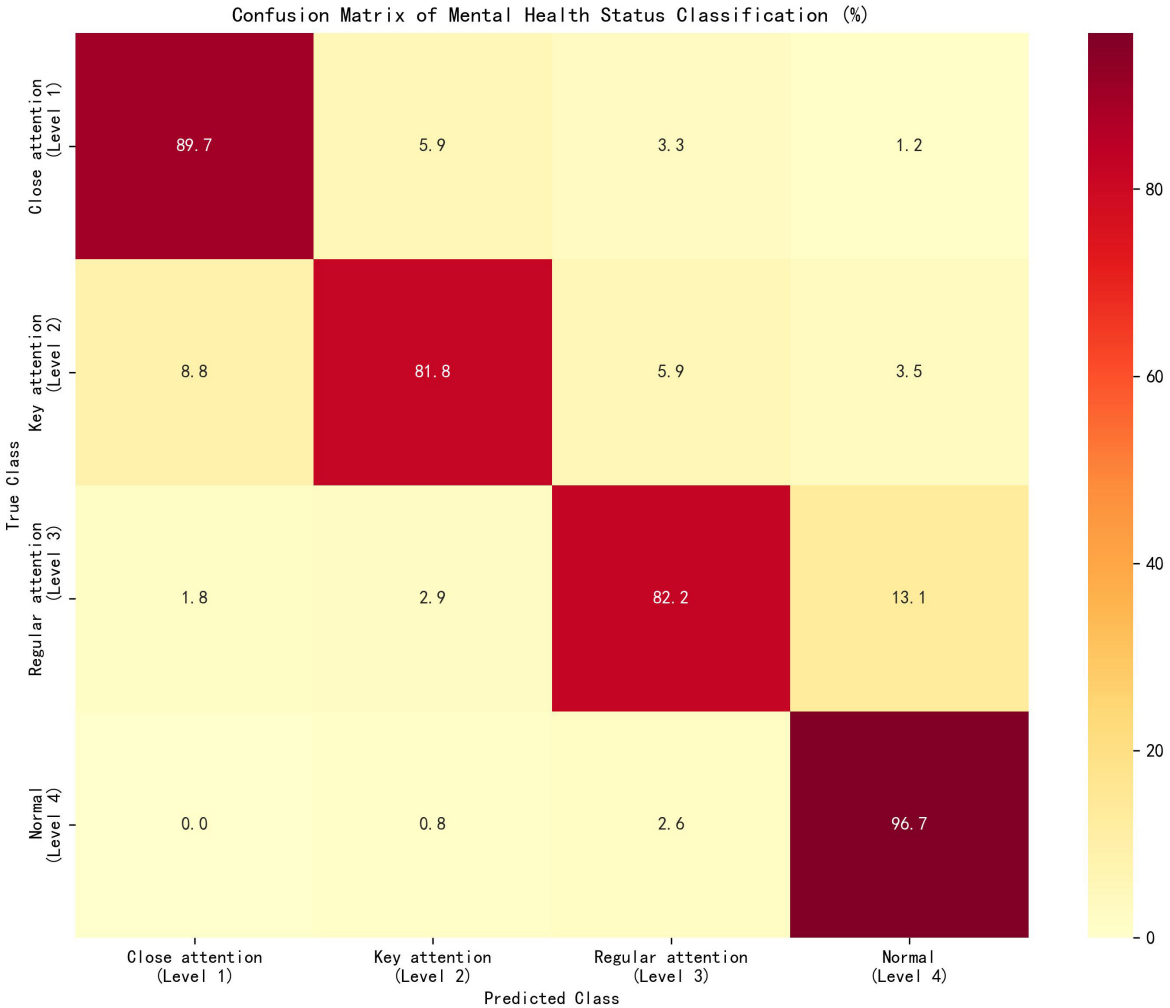
Confusion Matrix.

We visualized the classification performance using a row-normalized confusion matrix in Figure 3. The horizontal axis denotes the predicted classes, whereas the vertical axis represents the true classes. Each cell reports the proportion of samples from a given true class that were assigned to each predicted class. The off-diagonal entries are concentrated near the diagonal, suggesting that the majority of misclassifications occur between adjacent classes rather than between distant categories. This pattern implies that the model's errors are predominantly local confusions consistent with the ordinal proximity or similarity among neighboring classes. And to quantify performance under the ordinal label structure, we computed two ordinal-aware metrics. The model achieves a quadratic weighted kappa (QWK) of 0.914, indicating strong ordinal agreement between predictions and expert labels. In addition, the ordinal MAE is 0.153, meaning that the average prediction deviates from the true level by only about 0.15 levels. These results suggest that misclassifications are typically mild, which is consistent with the gradual transition between Levels 2 and 3. The population-adjusted evaluation metrics are presented in Table 4.

**Table 4:** Population-adjusted evaluation metrics.

Metric	Value
Population-adjusted accuracy	0.9613
Population-adjusted QWK	0.487
Population-adjusted ordinal MAE	0.0528

Because the four risk levels are extremely imbalanced in the full population (Level 1: 0.63%, Level 2: 1.29%, Level 3: 1.62%, Level 4: 96.46%), metrics computed on the class-balanced test set primarily reflect discriminative ability under controlled prevalence rather than expected operational performance under real base rates. To improve interpretability, we additionally report *population-adjusted* metrics by re-weighting each row of the row-normalized confusion matrix (Figure 3) according to the observed population prevalence, which yields the expected performance under the real-world class distribution. We emphasize that threshold-dependent quantities such as precision/PPV and false-positive burden can change materially when base rates shift; therefore, deployment should calibrate probabilities and select operating thresholds according to counseling capacity and acceptable false-alarm costs.

### 4.4 Multi-model comparison experiments

To comprehensively evaluate the proposed model's performance, we implemented multiple comparative models for experimental comparison. The comparative models include:

- Bert+SVM+Lasso: Combines BERT text features with SVM classifier and uses Lasso for feature selection
- BCL(Bert+CNN+Lasso): The complete model proposed in this paper
- Bert+CNN: Base model without Lasso
- SVM+Lasso: Baseline model using only static features
- RNN+LSTM: A dual-layer LSTM structure model based on a deep learning model (Amanat et al., 2022), specifically designed for text-based depression detection tasks

As shown in Table 5, our proposed BCL model outperforms all comparative models across evaluation metrics, achieving an accuracy of 0.7519, precision and recall of 0.7475 and 0.7490 respectively. This performance advantage is primarily reflected in several aspects:

**Table 5:** Multi-model comparison.

Model	Accuracy	Precision	Recall	F1 score
**BCL**	**0.7519**	0.7475	**0.7490**	**0.7478**
Bert+SVM+Lasso	0.7211	**0.7489**	0.7239	0.7363
Bert+CNN	0.6729	0.6776	0.6460	0.6614
RNN+LSTM	0.6521	0.6673	0.6219	0.6438
SVM+Lasso	0.5241	0.4990	0.5316	0.5148

Bold values indicate the best performance among all compared models for each metric (Accuracy, Precision, Recall, F1 score).

Comparing Bert+CNN+Lasso with its variant without static vectors (Bert+CNN), we can clearly observe that the Lasso module's feature selection and dimensionality reduction of static features bring an 8 percentage point performance improvement. This enhancement validates both the importance of student static features in psychological state classification and the effectiveness of our proposed feature fusion strategy. While improving precision, the model also achieves higher recall rates, indicating that feature fusion not only enhances overall accuracy but maintains balanced performance across metrics.

From an architectural perspective, the CNN structure demonstrates significant advantages. The Bert+CNN+Lasso model outperforms the SVM-structured Bert+SVM+Lasso by 3 percentage points, suggesting CNN's superior capability in capturing psychological state-related text features. This may be because key information in psychological counseling dialogues often manifests in local semantic patterns, which aligns well with CNN's local feature extraction capabilities.

Although the RNN+LSTM model demonstrated excellent depression detection capabilities in the original literature, it only achieved 0.6521 accuracy in our task. This performance difference may stem from task nature differences: depression detection primarily focuses on specific negative emotion expression patterns, with preprocessing specifically designed for depression detection, while psychological state grading requires understanding broader emotional expressions and psychological state characteristics. Additionally, our base model, Bert-Chinese, has been specifically pre-trained for Chinese language, providing more accurate Chinese semantic understanding, whereas LSTM-based methods require training from scratch and struggle to learn long-distance dependencies effectively on limited-scale datasets. Furthermore, the dual-layer LSTM structure's overemphasis on long-term dependency features may overlook immediate emotional expressions in dialogues, while CNN's local receptive field is better suited for capturing key phrases and contextual features in emotional expressions, thus achieving better results in this task.

While the SVM+Lasso model using only static features achieved an accuracy of just 0.5241, this baseline performance indicates that student static features indeed contain valuable predictive information. When combined with BERT text features, model performance improved significantly by 23%, not only confirming the importance of text features but also demonstrating good complementarity between static and text features. Additionally, this result showcases BERT pre-trained model's powerful capability in understanding psychological counseling dialogue texts and its good adaptability when combined with traditional features.

The experimental results strongly support our proposed model design. The three key design choices-CNN structure selection, static feature integration, and BERT feature application-are all validated by experimental data. Particularly noteworthy is the balanced performance achieving above 0.74 across accuracy, precision, and recall metrics, demonstrating the model's reliability in practical applications. This performance advantage is reflected not only in overall metrics but, more importantly, ensures stable identification capability across all risk levels, which is crucial for the practical application of psychological early warning systems.

### 4.5 Feature importance analysis

Through LASSO model's feature importance analysis, we identified key factors influencing student mental health state classification. The LASSO method, by introducing L1 regularization, enables automatic feature selection and importance estimation, with importance scores calculated from the sum of coefficient absolute values across model categories, providing good interpretability. Feature importance scores calculated from the sum of coefficient absolute values across model categories offer good interpretability, intuitively reflecting each feature's significance in the classification task. Larger coefficient absolute values indicate stronger predictive capability in distinguishing different mental health levels.

**Figure 4:**
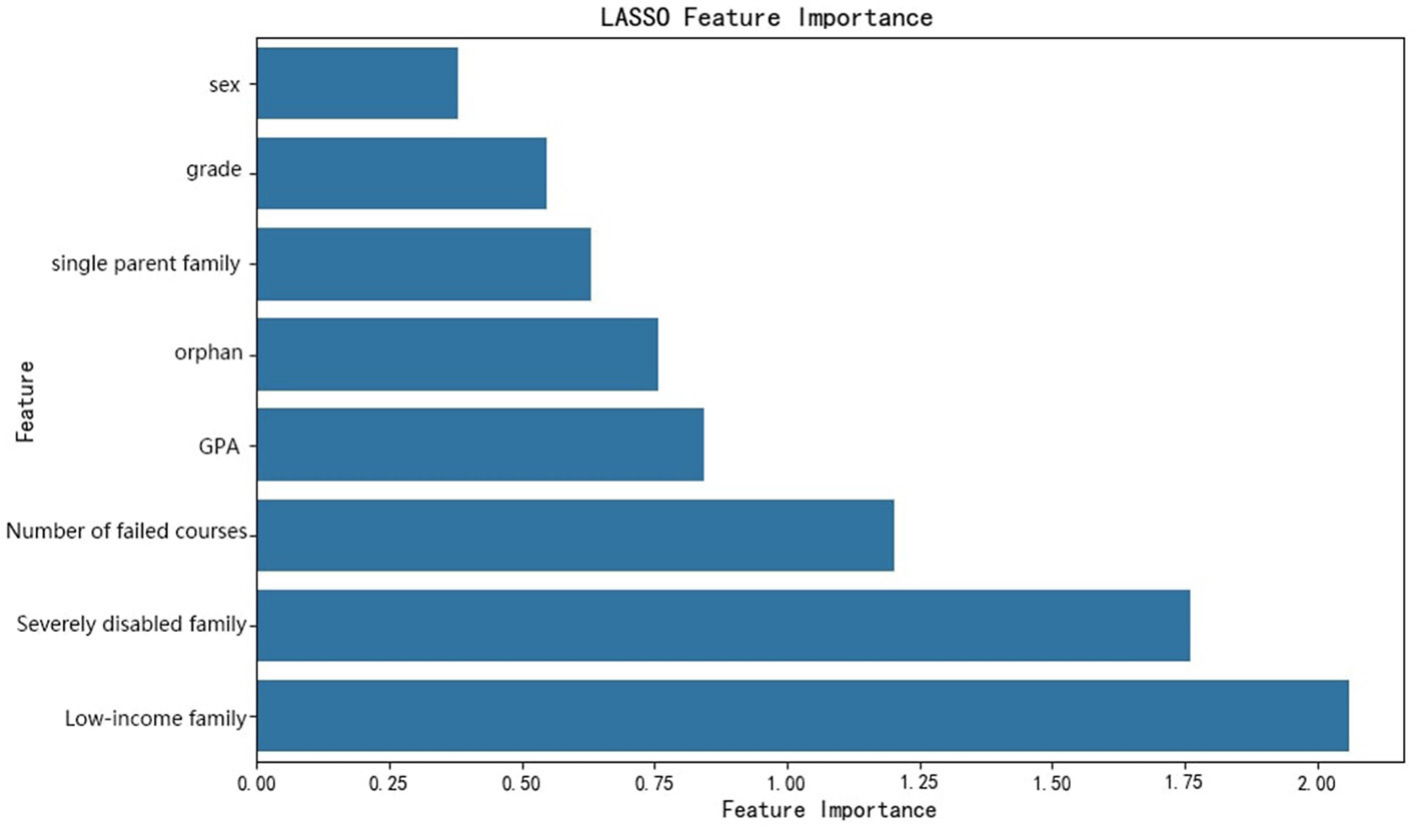
Feature importance analysis based on LASSO.

As shown in Figure 4, family economic status-related indicators played the most crucial role in prediction, with financial aid status (importance 2.0599) and severely disabled family (importance 1.7614) ranking first and second respectively. This suggests that family socioeconomic adversity is a prominent predictor in our dataset, consistent with prior evidence linking economic hardship to elevated mental health risk among university students (Sheldon et al., 2021). Among academic performance indicators, the number of failed courses (importance 1.2024) and GPA (importance 0.8432) also showed significant influence, confirming academic pressure as a crucial factor affecting student mental health (Wang et al., 2014).

Notably, special family backgrounds such as orphan status (importance 0.7586) and single-parent family (importance 0.6317), despite their relatively small sample sizes, demonstrated high predictive importance. While grade level (importance 0.5482) and gender (importance 0.3796) features show relatively lower importance, they still maintain predictive value, indicating the need to consider the effects of educational stage and gender differences in mental health interventions.

Additionally, in order to conduct interpretable analysis on text data, we performed a frequency-based lexical analysis to identify tokens that are over-represented in Level 1 dialogue records compared with Levels 2-4. All utterances were tokenized and normalized, after which word occurrences were counted within each comparison group. Group-wise term frequencies were then statistically compared, and only tokens showing significant between-group differences were retained for visualization.

**Figure 5:**
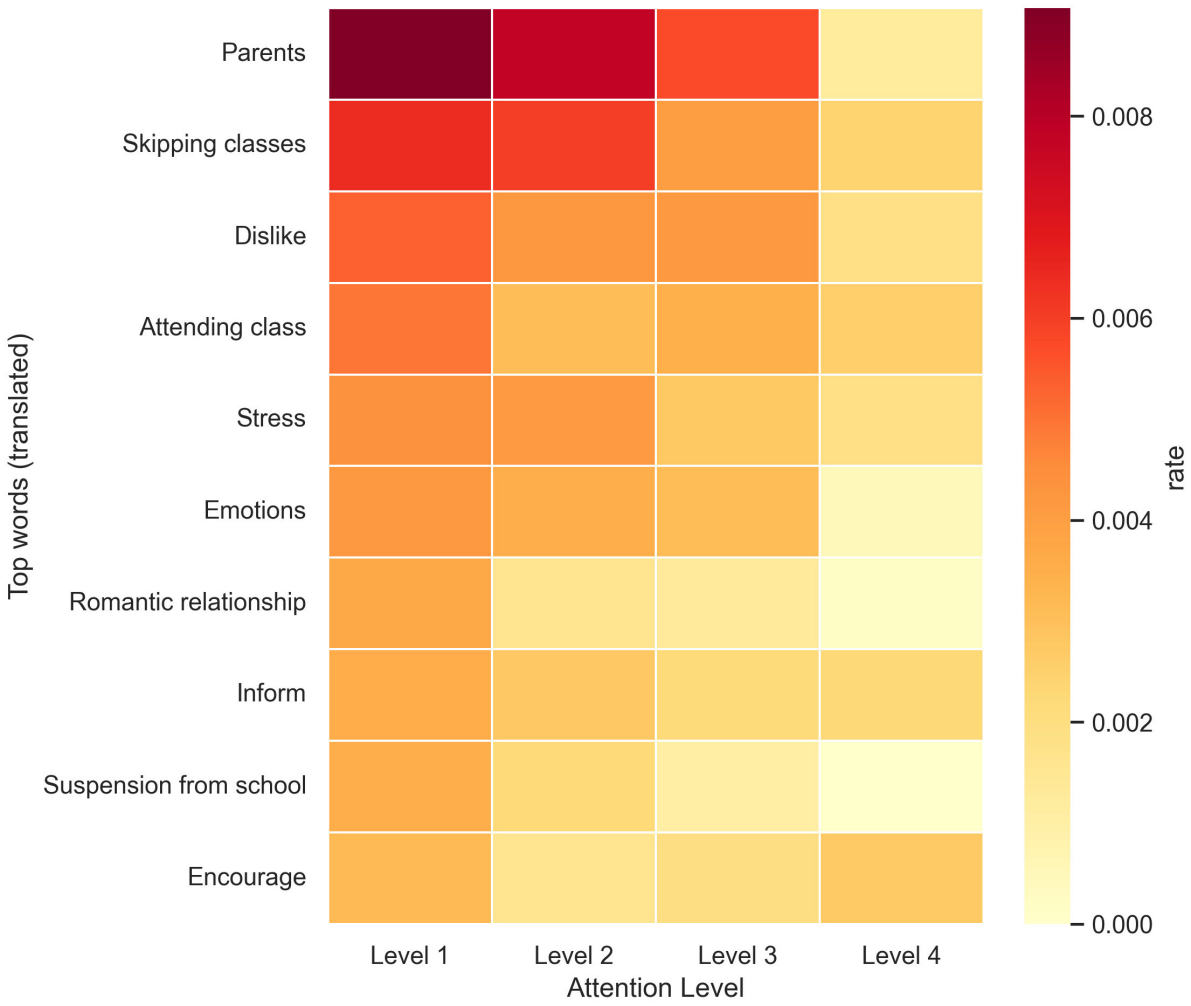
Heat map on Level 1.

**Figure 6:**
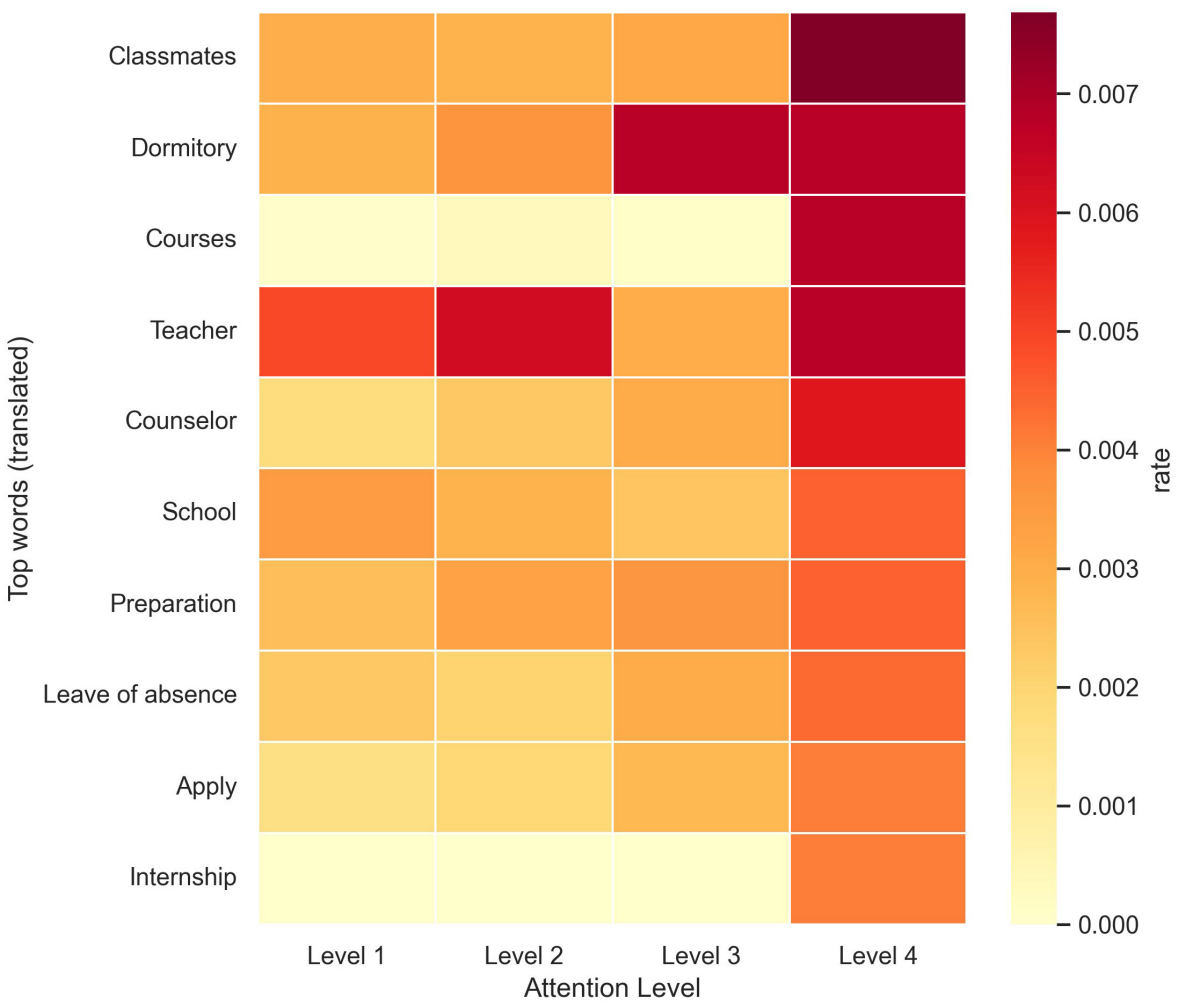
Heat map on Level 4.

Figures 5, 6 present heatmaps of tokens showing statistically significant frequency differences for the Level 1 and Level 4 dialogue records, respectively. Color intensity encodes standardized relative frequency, thereby highlighting distinctive lexical usage patterns. For the Level 1, the most discriminative words collectively reflect salient psychosocial stressors and behavioral/functional disruption commonly documented in high-risk interviews. In contrast, the low-risk group (Level 4) is characterized by words such as classmates, dormitory, and courses, indicating that Level 4 conversations more frequently focus on routine campus life, academic arrangements, and planning-related topics rather than crisis-oriented content.

These findings provide important references for optimizing mental health early warning systems: special attention should be paid to students from economically disadvantaged and special family backgrounds; timely follow-up is needed for students showing significant fluctuations in academic performance; and when providing mental health services, grade level and gender factors should be considered to develop more targeted intervention strategies.

At the same time, several of the static variables highlighted by the LASSO analysis (e.g., low-income family status, orphan status, single-parent family, severely disabled family) reflect socioeconomic vulnerability and are ethically sensitive. Any real-world use of such predictors must therefore carefully assess subgroup error patterns and avoid stigmatizing or discriminatory interpretations. In this study, the model is evaluated as a research prototype and not deployed as an automated decision-making tool; systematic fairness analysis and bias mitigation are important directions for future work.

## 5 Conclusion

This research addresses the crucial topic of mental health early warning in higher education by proposing a deep learning classification model based on multimodal data. The model effectively integrates students' static feature data and dialogue text data to achieve automated classification of students' mental health states. In terms of data processing, we established a high-quality dataset of 600 students, encompassing structured personal attribute features and unstructured dialogue text data. Through feature engineering and data preprocessing, we ensured data quality and model training effectiveness. Feature-importance analysis indicated that family socioeconomic/background variables were the most influential predictors, followed by academic performance indicators.

From an architectural perspective, the proposed model employs a theme-specific ensemble strategy that aggregates predictions across five counseling themes, improving classification accuracy and robustness through the collaboration of multiple base classifiers. On the class-balanced test set, BCL achieves an overall accuracy of 0.752. At the category level, it attains accuracies of 0.897 for the highest-risk group (Level 1) and 0.967 for the lowest-risk group (Level 4), while maintaining a high recall for Level 1. This pattern of performance is well aligned with the operational goals of early warning systems, which require prioritizing the detection of high-risk students without generating excessive false alarms for low-risk students.

However, the research also identified several directions worthy of further exploration. The model's classification performance for medium risk levels (key attention and regular attention categories) was relatively weaker, indicating the need to further optimize the model's ability to identify boundary cases. Current feature extraction methods mainly focus on explicit features; future work could consider incorporating more implicit features, such as student behavioral trajectory data and social network data, to provide more comprehensive mental health status assessment. Additionally, there is room for improvement in model interpretability, particularly in explaining specific prediction rationales, which is crucial for intervention decision-making in practical applications.

Looking ahead, we envision several important trends in data-driven university mental health early warning. Future systems are likely to rely on larger, multi-institutional, and longitudinal datasets that integrate counseling records with multimodal behavioral signals, enabling more stable risk estimation over time and across diverse student populations. Advances in large language models and multimodal representation learning also create opportunities to move beyond surface-level text features toward deeper modeling of conversational context, discourse structure, and intent, potentially supporting not only risk stratification but also personalized feedback and intervention design under strict human oversight. In parallel, there is a growing need for continuously updated, human–AI collaborative decision-support tools, in which algorithms prioritize cases and provide transparent rationales, while final judgments and interventions remain under the control of trained mental health professionals. Finally, future research will need to place greater emphasis on fairness, transparency, and privacy-preserving analytics to ensure that early warning systems are ethically acceptable and trusted by students, counselors, and institutions.

Based on the current findings, an immediate practical direction is to embed the proposed early warning model into a comprehensive student support system that links automated risk stratification with real-time monitoring dashboards, alert delivery to counselors, appointment management, and follow-up tracking. By progressively extending such systems in line with emerging trends in multimodal learning, privacy-aware analytics, and human–AI collaboration, universities may be able to substantially increase both the coverage and precision of mental health services while maintaining ethical and professional standards.

## Data Availability

The data analyzed in this study is subject to the following licenses/restrictions: The dataset used in this study consists of real mental health records collected by the university's counseling center. Due to the sensitive nature of the data and the involvement of student privacy, access to the dataset is strictly restricted and cannot be disclosed publicly. Data sharing is not permitted in order to protect personal and confidential information of the students. Requests to access these datasets should be directed to Fu Li, 42582@hdu.edu.cn.
